# Prevalence of Autism Spectrum Disorder Among Children Aged 8 Years — Autism and Developmental Disabilities Monitoring Network, 11 Sites, United States, 2016

**DOI:** 10.15585/mmwr.ss6904a1

**Published:** 2020-03-27

**Authors:** Matthew J. Maenner, Kelly A. Shaw, Jon Baio, EdS, Anita Washington, Mary Patrick, Monica DiRienzo, Deborah L. Christensen, Lisa D. Wiggins, Sydney Pettygrove, Jennifer G. Andrews, Maya Lopez, Allison Hudson, Thaer Baroud, Yvette Schwenk, Tiffany White, Cordelia Robinson Rosenberg, Li-Ching Lee, Rebecca A Harrington, Margaret Huston, Amy Hewitt, PhD-, Amy Esler, Jennifer Hall-Lande, Jenny N. Poynter, Libby Hallas-Muchow, John N. Constantino, Robert T. Fitzgerald, Walter Zahorodny, Josephine Shenouda, Julie L. Daniels, Zachary Warren, Alison Vehorn, Angelica Salinas, Maureen S. Durkin, Patricia M. Dietz

**Affiliations:** ^1^National Center on Birth Defects and Developmental Disabilities, CDC, Atlanta, Georgia; ^2^University of Arizona, Tucson; ^3^University of Arkansas for Medical Sciences, Little Rock; ^4^Colorado Department of Public Health and Environment, Denver; ^5^University of Colorado School of Medicine, Department of Pediatrics, Aurora; ^6^Johns Hopkins University, Baltimore, Maryland; ^7^University of Minnesota, Minneapolis; ^8^Washington University in St. Louis, Missouri; ^9^Rutgers New Jersey Medical School, Newark; ^10^University of North Carolina, Chapel Hill; ^11^Vanderbilt University Medical Center, Nashville, Tennessee; ^12^University of Wisconsin, Madison

## Abstract

**Problem/Condition:**

Autism spectrum disorder (ASD).

**Period Covered:**

2016.

**Description of System:**

The Autism and Developmental Disabilities Monitoring (ADDM) Network is an active surveillance program that provides estimates of the prevalence of ASD among children aged 8 years whose parents or guardians live in 11 ADDM Network sites in the United States (Arizona, Arkansas, Colorado, Georgia, Maryland, Minnesota, Missouri, New Jersey, North Carolina, Tennessee, and Wisconsin). Surveillance is conducted in two phases. The first phase involves review and abstraction of comprehensive evaluations that were completed by medical and educational service providers in the community. In the second phase, experienced clinicians who systematically review all abstracted information determine ASD case status. The case definition is based on ASD criteria described in the *Diagnostic and Statistical Manual of Mental Disorders, Fifth Edition*.

**Results:**

For 2016, across all 11 sites, ASD prevalence was 18.5 per 1,000 (one in 54) children aged 8 years, and ASD was 4.3 times as prevalent among boys as among girls. ASD prevalence varied by site, ranging from 13.1 (Colorado) to 31.4 (New Jersey). Prevalence estimates were approximately identical for non-Hispanic white (white), non-Hispanic black (black), and Asian/Pacific Islander children (18.5, 18.3, and 17.9, respectively) but lower for Hispanic children (15.4). Among children with ASD for whom data on intellectual or cognitive functioning were available, 33% were classified as having intellectual disability (intelligence quotient [IQ] ≤70); this percentage was higher among girls than boys (40% versus 32%) and among black and Hispanic than white children (47%, 36%, and 27%, respectively). Black children with ASD were less likely to have a first evaluation by age 36 months than were white children with ASD (40% versus 45%). The overall median age at earliest known ASD diagnosis (51 months) was similar by sex and racial and ethnic groups; however, black children with IQ ≤70 had a later median age at ASD diagnosis than white children with IQ ≤70 (48 months versus 42 months).

**Interpretation:**

The prevalence of ASD varied considerably across sites and was higher than previous estimates since 2014. Although no overall difference in ASD prevalence between black and white children aged 8 years was observed, the disparities for black children persisted in early evaluation and diagnosis of ASD. Hispanic children also continue to be identified as having ASD less frequently than white or black children.

**Public Health Action:**

These findings highlight the variability in the evaluation and detection of ASD across communities and between sociodemographic groups. Continued efforts are needed for early and equitable identification of ASD and timely enrollment in services.

## Introduction

Autism spectrum disorder (ASD) is a developmental disability characterized by persistent impairments in social interaction and the presence of restricted, repetitive patterns of behaviors, interests, or activities (*1*). CDC has been tracking the prevalence of ASD since 1996, beginning with children in metropolitan Atlanta, Georgia (*2*). Subsequently, CDC established the Autism and Developmental Disabilities Monitoring (ADDM) Network, which has reported ASD prevalence in multiple communities in even-numbered years since 2000.

The previous ADDM Network ASD prevalence estimate was 16.8 per 1,000 (one in 59) children aged 8 years in 2014 (*3*). This is approximately 2.5 times higher than the first ADDM Network ASD prevalence estimates of 6.7 (one in 150) from 2000 and 2002 (*4*–*7*). Findings from each surveillance year between 2000 and 2014 have included variability and disparities in the prevalence of ASD (*3*–*5*,*8*–*11*). In contrast to other developmental disabilities (*12*–*15*), the ADDM Network reported higher ASD prevalence among more socioeconomically advantaged groups and among children classified as non-Hispanic white (white) than among other groups (*16*,*17*). Overall, the magnitude of prevalence differences by race and ethnicity has declined in recent years (*3*,*17*). Reduction of these disparities might indicate progress toward enhanced detection of ASD among all children.

Timely evaluation and identification of ASD among young children continue to be important public health goals (*18*,*19*) because evidence links early treatment and services for ASD with improved outcomes (*20*–*23*). Although greater numbers of children are identified as having ASD over time, previous ADDM Network findings suggest little overall change in the median age at ASD diagnosis (range: 50–56 months), and fewer than half of children with ASD had a record of a developmental evaluation by age 36 months (*3*–*5*,*8*–*11*). However, considerable variability has been reported between communities in both ASD prevalence and the ages at which ASD is diagnosed.

This report provides the latest available data on ASD prevalence among children aged 8 years living in ADDM Network sites in 2016, including variations in prevalence by site and demographic characteristics, median ages when children are evaluated and ASD is diagnosed, and co-occurrence of intellectual disability. Pediatric health care providers, educators, researchers, service providers, and policymakers can use these data to anticipate service needs in their communities and help develop policies that ensure early and comprehensive identification of ASD.

## Methods

### Surveillance Sites and Procedures

The ADDM Network was composed of 11 sites for surveillance year 2016 (Arizona, Arkansas, Colorado, Georgia, Maryland, Minnesota, Missouri, New Jersey, North Carolina, Tennessee, and Wisconsin). Children included in ADDM surveillance year 2016 were born in 2008 and had a parent or guardian who lived in one of 11 surveillance sites in 2016. Each site selected a portion of its state (except Arkansas, which included the entire state) to monitor ASD among children aged 8 years in 2016. All sites functioned as public health authorities under the Health Insurance Portability and Accountability Act of 1996 Privacy Rule and met applicable local institutional review board, privacy, and confidentiality requirements under 45 CFR 46 (*24*). The racial and ethnic composition of populations in ADDM Network sites is provided (Supplementary Table 1, https://stacks.cdc.gov/view/cdc/85386).

### Case Ascertainment and Surveillance Case Definition

The ADDM Network uses a multiple-source, records-based surveillance methodology developed by CDC’s Metropolitan Atlanta Developmental Disabilities Surveillance Program (*2*,*24*). The ADDM Network ASD surveillance methodology is a two-phase process that has been described previously (*3*). In brief, in the first phase, ADDM Network staff review records from medical, education, and service providers (e.g., autism specialty clinics or intervention providers) in the community after requesting records that include various billing codes from the *International Classification of Disease, Ninth Revision* (ICD-9) or *International Classification of Diseases, Tenth Revision* (ICD-10) or special education exceptionalities (Supplementary Table 2, https://stacks.cdc.gov/view/cdc/85386). If any record contains an indication of ASD, the child’s evaluations and other information (e.g., intelligence quotient [IQ] tests) are abstracted and compiled from all available sources in the community. Although all ADDM Network sites use records from medical and service providers, not all sites have complete access to education records.

In the second phase, an ADDM Network clinician reviews the deidentified, compiled record for each child to determine ASD case status. The ADDM Network ASD case definition is based on the *Diagnostic and Statistical Manual of Mental Disorders, Fifth Edition* (DSM-5), and the process for scoring the features of the surveillance case definition have been described previously (*3*,*25*,*26*). ADDM Network clinicians might assign ASD case status if documented evidence satisfies the behavioral criteria for the ASD case definition, or if the child has an established ASD diagnosis. ADDM Network clinicians might decide a child who otherwise meets ASD surveillance criteria should not be included as a case because of insufficient or conflicting information or if other conditions better account for the child’s symptoms. Another clinician performs a secondary review if the first reviewer indicates uncertainty. To monitor interrater reliability, 10% of records were randomly selected for an independent review (ASD case status kappa = 0.89) (Supplementary Table 3, https://stacks.cdc.gov/view/cdc/85386). At most ADDM Network sites, clinicians also applied the previous ASD case definition based on the *Diagnostic and Statistical Manual of Mental Disorders, Fourth Edition, Text Revision* (DSM-IV-TR) for at least a portion of the children with abstracted information.

### Additional Data Sources and Variable Definitions

Population denominators were obtained from the National Center for Health Statistics vintage 2018 bridged-race postcensal population estimates for 2016 (*27*). For study areas comprising subcounty school districts, a standardization process using public school enrollment counts was used to adjust the population estimates (Supplementary Methods, https://stacks.cdc.gov/view/cdc/85386). Each site linked each child to birth certificate information from their state. When successful, this linkage indicates which children were born in the state that they lived in at age 8 years and provides additional demographic information. Information about race and ethnicity came from information abstracted from the medical or education records, which was augmented by data from birth certificates and data from administrative or billing information. Children with race coded as “other” or “multiracial” were excluded from race-specific estimates, as were American Indian/Alaskan Native children because of small numbers.

Age at first developmental evaluation on record was based on each child’s abstracted evaluation information and restricted to children born in the state (or ADDM Network surveillance area in Minnesota) where the ADDM Network site is located. Age at first ASD diagnosis was based on the age of a child when an examiner recorded an ASD diagnostic statement or noted the child’s age when another provider previously diagnosed ASD. Intellectual disability status was based on IQ scores ≤70 on a child’s most recent test available through 2016. A child without an IQ score also could be classified as having intellectual disability on the basis of an examiner’s statement of intellectual disability in a developmental evaluation. Children were considered to have community-identified ASD if their records contained any of the following: 1) a diagnostic statement from a qualified professional of autistic disorder, pervasive developmental disorder not otherwise specified (PDD-NOS), Asperger disorder, or ASD; 2) any ASD ICD billing code at any time from birth through 2016; or 3) receipt of (or met eligibility for) special education services under the autism classification in public school.

### Analytic Methods

Prevalence was calculated as the number of children with ASD per 1,000 children aged 8 years in the defined population or subgroup. Overall prevalence estimates included all children identified with ASD. Results for the combined (overall) total include data from all sites unless otherwise noted. Ninety-five percent confidence intervals (CIs) for prevalence, proportions, and prevalence ratios were calculated using the Wilson score method. Pearson chi-square tests were performed for comparison of proportions, and the Mantel-Haenszel (Woolf) test of homogeneity was used to compare prevalence ratios across sites. Permutation tests were conducted to test differences in medians. Statistical tests with p values <0.05 were considered statistically significant, as were 95% CIs that excluded 1.0 for prevalence ratios. Cumulative incidence of ASD diagnoses was calculated as the total children with ASD diagnosed during or before a given month of age, divided by the total population of children aged 8 years in the surveillance area. R software (version 3.5.3; R Foundation) and additional packages were used to conduct analyses. Additional information about the statistical software is available (Supplementary Table 4, https://stacks.cdc.gov/view/cdc/85386).

## Results

### ASD Prevalence

The combined ASD prevalence, with data from all 11 sites, was 18.5 per 1,000 (one in 54) children aged 8 years. ASD prevalence ranged from 13.1 per 1,000 children aged 8 years (one in 76) in Colorado to 31.4 per 1,000 children aged 8 years (one in 32) in New Jersey (Table 1). The estimate for New Jersey was higher than for every other ADDM Network site. Two sites with limited or no access to education records had the lowest ASD prevalence estimates (Colorado [13.1] and Missouri [13.6]). ASD prevalence among boys was higher than among girls (29.7 versus 6.9). The combined male-to-female prevalence ratio was 4.3:1; site-specific ratios ranged from 3.4:1 to 4.7:1, with little evidence of heterogeneity by site.

**TABLE 1:** Prevalence* of autism spectrum disorder among children aged 8 years, overall and by sex — Autism and Developmental Disabilities Monitoring Network, 11 sites, United States, 2016

Site	Overall^†^				Male	Female	Male-to-female prevalence ratio (95% CI)^§^
	Description of surveillance area	No. with ASD	Total population	Prevalence (95% CI)	Prevalence (95% CI)	Prevalence (95% CI)	
Arizona	Part of one county in metropolitan Phoenix^†^	282	17,656	16.0 (14.2–17.9)	25.4 (22.4–28.9)	6.0 (4.6–7.9)	4.2 (3.1–5.7)
Arkansas	All 75 counties in Arkansas	606	40,225	15.1 (13.9–16.3)	24.3 (22.3–26.5)	5.4 (4.5–6.5)	4.5 (3.7–5.5)
Colorado	Seven counties in metropolitan Denver	537	40,874	13.1 (12.1–14.3)	21.2 (19.3–23.2)	4.7 (3.9–5.8)	4.5 (3.6–5.6)
Georgia	Two counties in metropolitan Atlanta	456	24,113	18.9 (17.3–20.7)	30.4 (27.5–33.6)	7.1 (5.7–8.7)	4.3 (3.4–5.4)
Maryland	One county in metropolitan Baltimore	192	9,993	19.2 (16.7–22.1)	30.1 (25.8–35.2)	7.8 (5.7–10.7)	3.9 (2.7–5.5)
Minnesota	Parts of two counties including Minneapolis–St. Paul	313	13,728	22.8 (20.4–25.4)	36.3 (32.1–41.0)	9.2 (7.2–11.8)	3.9 (3.0–5.2)
Missouri	Two counties in metropolitan St. Louis	213	15,635	13.6 (11.9–15.6)	21.1 (18.1–24.5)	6.2 (4.6– 8.1)	3.4 (2.5–4.7)
New Jersey	Four counties including metropolitan Newark	1,036	33,031	31.4 (29.5–33.3)	50.0 (46.8–53.4)	12.0 (10.4–13.8)	4.2 (3.6–4.9)
North Carolina	Four counties in central North Carolina	489	19,291	25.3 (23.2–27.7)	41.2 (37.5–45.3)	8.7 (7.0–10.8)	4.7 (3.7–6.0)
Tennessee	11 counties in middle Tennessee	405	25,839	15.7 (14.2–17.3)	25.5 (22.9–28.3)	5.5 (4.4–7.0)	4.6 (3.6–6.0)
Wisconsin	10 counties in southeastern Wisconsin	579	35,034	16.5 (15.2–17.9)	26.3 (24.0–28.7)	6.3 (5.2–7.6)	4.2 (3.4–5.2)
**Total**		**5,108**	**275,419**	**18.5 (18.0–19.1)**	**29.7 (28.8–30.6)**	**6.9 (6.5–7.4)**	**4.3 (4.0–4.6)**

**Abbreviations:** ASD = autism spectrum disorder; CI = confidence interval.

* Per 1,000 children aged 8 years.

^†^ All children are included in the total regardless of sex or race/ethnicity.

^§^ Wilson score 95% CIs exclude 1.0 in all sites, indicating significantly higher prevalence among males than among females.

Comparisons between surveillance years 2014 and 2016 are available (Supplementary Table 5, https://stacks.cdc.gov/view/cdc/85386). Among five sites that used the DSM-5–based case definition within the same geographic areas in 2014 and 2016, Maryland and Tennessee reported similar ASD prevalence in both years, whereas Arkansas, New Jersey, and Wisconsin reported increases. In the subset of children whose records also were reviewed using ADDM Network DSM-IV-TR ASD criteria, the DSM-IV-TR criteria classified 2% more ASD cases than DSM-5 criteria (Supplementary Table 6, https://stacks.cdc.gov/view/cdc/85386).

Overall ASD prevalence per 1,000 children aged 8 years was similar among white and non-Hispanic black (black) children (18.5 and 18.3, respectively) (Table 2). The white-to-black ASD prevalence ratio for the combined ADDM Network was 1.0; however, the prevalence ratio for white to black children was >1.0 in two ADDM Network sites (Arkansas and New Jersey). ASD prevalence among Asian/Pacific Islander children was 17.9 and similar to that among white and black children. ASD prevalence among Hispanic children was 15.4, which was lower than the prevalence among white and black children (white-to-Hispanic prevalence ratio and black-to-Hispanic prevalence ratio: 1.2). Numerator and denominator counts by race and ethnicity are available (Supplementary Table 7, https://stacks.cdc.gov/view/cdc/85386).

**TABLE 2:** Prevalence* of autism spectrum disorder among children aged 8 years, by race/ethnicity — Autism and Developmental Disabilities Monitoring Network, 11 sites, United States, 2016

Site	Non-Hispanic white	Non-Hispanic black	Hispanic	Asian/Pacific Islander^†^	Prevalence ratios		
	Prevalence (95% CI)	Prevalence (95% CI)	Prevalence (95% CI)	Prevalence (95% CI)	Non-Hispanic white to non-Hispanic black (95% CI)	Non-Hispanic white to Hispanic (95% CI)	Non-Hispanic black to Hispanic (95% CI)
Arizona	18.8 (16.0–22.0)	12.3 (7.6–19.9)	12.4 (10.1–15.3)	11.1 (5.6–21.8)	1.5 (0.9–2.5)	1.5 (1.2–2.0)^§^	1.0 (0.6–1.7)
Arkansas	16.6 (15.1–18.2)	12.1 (9.9–14.8)	9.6 (7.3–12.7)	18.6 (11.8–29.1)	1.4 (1.1–1.7)^§^	1.7 (1.3–2.3)^§^	1.3 (0.9–1.8)
Colorado	13.0 (11.6–14.6)	15.4 (11.5–20.7)	10.3 (8.8–12.1)	8.3 (5.2–13.3)	0.8 (0.6–1.2)	1.3 (1.0–1.5)^§^	1.5 (1.1–2.1)^§^
Georgia	18.9 (15.9–22.5)	19.7 (17.1–22.6)	11.3 (8.9–14.4)	23.0 (17.3–30.6)	1.0 (0.8–1.2)	1.7 (1.2–2.3)^§^	1.7 (1.3–2.3)^§^
Maryland	16.8 (13.5–20.8)	19.6 (15.5–24.7)	13.6 (7.8–23.6)	17.9 (10.5–30.4)	0.9 (0.6–1.2)	1.2 (0.7–2.2)	1.4 (0.8–2.6)
Minnesota	24.6 (20.9–28.9)	25.8 (21.2–31.5)	17.6 (12.8–24.3)	16.3 (11.7–22.6)	1.0 (0.7–1.2)	1.4 (1.0–2.0)	1.5 (1.0–2.1)^§^
Missouri	15.2 (12.8–18.2)	11.6 (9.2–14.6)	3.8 (1.3–11.1)	4.2 (1.4–12.4)	1.3 (1.0–1.8)	4.0 (1.4–11.9)^§^	3.1 (1.0–9.2)^§^
New Jersey	33.4 (30.5–36.6)	26.3 (22.8–30.2)	29.8 (26.7–33.2)	26.9 (20.7–35.0)	1.3 (1.1–1.5)^§^	1.1 (1.0–1.3)	0.9 (0.7–1.1)
North Carolina	23.3 (20.6–26.3)	27.9 (23.2–33.4)	19.3 (15.1–24.6)	28.2 (20.8–38.2)	0.8 (0.7–1.0)	1.2 (0.9–1.6)	1.4 (1.1–2.0)^§^
Tennessee	15.6 (13.8–17.6)	17.4 (14.2–21.3)	11.1 (8.1–15.1)	14.9 (8.7–25.3)	0.9 (0.7–1.1)	1.4 (1.0–2.0)^§^	1.6 (1.1–2.3)^§^
Wisconsin	17.0 (15.3–18.9)	13.8 (11.3–16.9)	15.4 (12.6–18.7)	15.5 (10.5–22.8)	1.2 (1.0–1.5)	1.1 (0.9–1.4)	0.9 (0.7–1.2)
**Total**	**18.5 (17.9–19.3)**	**18.3 (17.2–19.4)**	**15.4 (14.4–16.4)**	**17.9 (15.9–20.1)**	**1.0 (0.9–1.1)**	**1.2 (1.1–1.3)** ^§^	**1.2 (1.1–1.3)** ^§^

**Abbreviation:** CI = confidence interval.

* Per 1,000 children aged 8 years.

^†^ Because of small sample size relative to other groups, prevalence ratios are not shown for Asians/Pacific Islanders. Overall, the Hispanic-to-Asian/Pacific Islander prevalence ratio is statistically significant (prevalence ratio: 0.9; Wilson score: 95% CI = 0.8–0.99), but the non-Hispanic white-to-Asian/Pacific Islander and non-Hispanic black-to-Asian/Pacific Islander prevalence ratios are not statistically different from 1.0. Counts for the numerator and denominator are available (Supplementary Table 8, [https://stacks.cdc.gov/view/cdc/85386]).

^§^ Wilson score 95% CIs exclude 1.0, indicating significantly different prevalence between groups.

### Co-Occurring Intellectual Disability

**FIGURE 1:**
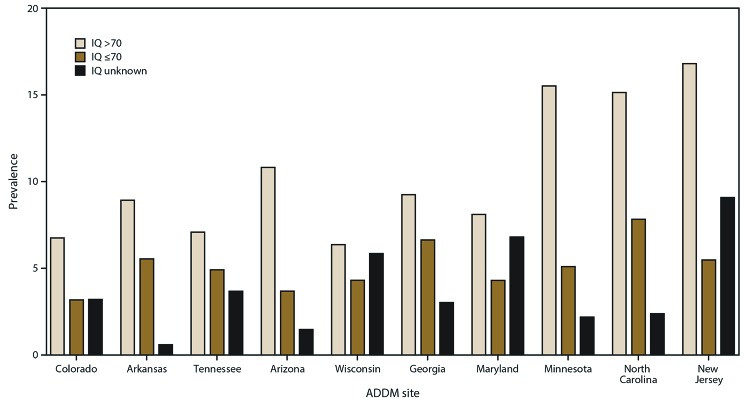
Prevalence* of autism spectrum disorder among children aged 8 years, by most recent intelligence quotient score and site — Autism and Developmental Disabilities Monitoring Network, 10 sites,^†^ United States, 2016 **Abbreviations:** ADDM = Autism and Developmental Disabilities Monitoring Network; IQ = intelligence quotient. * Per 1,000 children aged 8 years. ^†^ Missouri is not included because it did not collect IQ information on at least 60% of children with ASD. The total numbers of children with ASD, by site: n = 537 (Colorado), n = 606 (Arkansas), n = 405 (Tennessee), n = 282 (Arizona), n = 579 (Wisconsin), n = 456 (Georgia), n = 192 (Maryland), n = 313 (Minnesota), n = 489 (North Carolina), and n = 1,036 (New Jersey).

Ten of the 11 ADDM Network sites collected information on intellectual functioning for at least 60% of children meeting the ASD case definition (range: 65% [Maryland and Wisconsin] to 96% [Arkansas]). Similar proportions of cases among boys and girls had information on intellectual ability (80% versus 78%), as did white and black children (81% versus 79%). Across states, greater absolute variability was reported in the prevalence of ASD without intellectual disability than ASD with intellectual disability at the 10 sites (Figure 1).

Among children meeting ASD case status who had IQ information, 33% were classified as having intellectual disability (IQ ≤70) at their most recent test or examination, 24% had an IQ in the borderline range (IQ 71–85), and 42% had an IQ in the average or higher range (IQ >85) (Table 3). The percentage of children with co-occurring intellectual disability varied by site (range: 25% [New Jersey] to 42% [Georgia]). Overall, a higher percentage of girls than boys was classified as having intellectual disability (40% versus 32%), and black and Hispanic children were more likely than white children to be classified as having intellectual disability (47%, 36%, and 27%, respectively) (Supplementary Figures, https://stacks.cdc.gov/view/cdc/85386).

**TABLE 3:** Availability and distribution of intelligence quotient scores among children aged 8 years with autism spectrum disorder — Autism and Developmental Disabilities Monitoring Network, 10 sites,^†^ United States, 2016

Site	Total no. with ASD	With IQ information	Cognitive level*		
		No. (%)	IQ <70 (%)	IQ 71–85 (%)	IQ >85 (%)
Arizona	282	256 (90.8)	25.4	30.1	44.5
Arkansas	606	582 (96.0)	38.3	23.0	38.1
Colorado	537	406 (75.6)	32.0	22.2	45.8
Georgia	456	383 (84.0)	41.8	20.9	37.1
Maryland	192	124 (64.6)	34.7	25.8	38.7
Minnesota	313	283 (90.4)	24.7	19.8	54.1
New Jersey	1,036	736 (71.0)	24.6	27.9	47.4
North Carolina	489	443 (90.6)	34.1	20.8	44.9
Tennessee	405	310 (76.5)	41.0	30.3	27.7
Wisconsin	579	374 (64.6)	40.4	21.7	38.0
**Total**	**4,895**	**3,897 (79.6)**	**33.4**	**24.1**	**42.1**

**Abbreviation:** ASD = autism spectrum disorder; IQ = intelligence quotient.

* Levels of intellectual functioning might not always sum to exactly 100% because 15 children (three in Arkansas, one in Georgia, one in Maryland, four in Minnesota, one in North Carolina, one in New Jersey, and three in Tennessee) had evidence of IQ >70 (i.e., examiner statement) but without sufficient detail to be assigned to either the IQ 71–85 or IQ >85 categories.

^†^ Missouri is not included because it did not collect IQ information on at least 60% of children with ASD.

### Age at First Evaluation and ASD Diagnosis

Among 3,981 children aged 8 years with ASD who were born in the state of residence, 44% were evaluated by age 36 months, with wide variation across ADDM Network sites (range: 33% [Arkansas] to 62% [North Carolina]) (Table 4). The median age at first evaluation ranged from 29 months (North Carolina) to 46 months (Arkansas). A higher percentage of girls was evaluated by age 36 months than boys (48% versus 43%) (Table 4). The majority of children with ASD and IQ ≤70 (58%) were evaluated by age 36 months, compared with 38% of children with IQ >70. The percentage of children with ASD evaluated by age 36 months varied by race and ethnicity: 45% among white children, 43% among Hispanic children, and 40% among black children.

**TABLE 4:** Number and percentage of children aged 8 years with autism spectrum disorder who received a comprehensive evaluation by a qualified professional at age ≤36 months, 37–48 months, or >48 months and the median age at first evaluation, by site and selected characteristics — Autism and Developmental Disabilities Monitoring Network, 11 sites, United States, 2016

Site/Characteristic	Total no. of children with ASD and a linked birth certificate	Youngest age when child first received a comprehensive evaluation			Median age (mos) at first evaluation
		≤36 mos	37–48 mos	>48 mos	
		No. (%)	No. (%)	No. (%)	
**Site**					
Arizona	218	83 (38.1)	53 (24.3)	82 (37.6)	43.0
Arkansas	481	157 (32.6)	105 (21.8)	219 (45.5)	46.0
Colorado	388	186 (47.9)	60 (15.5)	142 (36.6)	37.5
Georgia	337	125 (37.1)	79 (23.4)	133 (39.5)	43.0
Maryland	172	83 (48.3)	37 (21.5)	52 (30.2)	38.0
Minnesota	246	96 (39.0)	48 (19.5)	102 (41.5)	44.5
Missouri	147	58 (39.5)	34 (23.1)	55 (37.4)	41.0
New Jersey	816	355 (43.5)	163 (20.0)	298 (36.5)	39.5
North Carolina	371	231 (62.3)	42 (11.3)	98 (26.4)	29.0
Tennessee	314	113 (36.0)	69 (22.0)	132 (42.0)	45.0
Wisconsin	491	247 (50.3)	87 (17.7)	157 (32.0)	36.0
**Characteristic**					
Sex					
Female	730	352 (48.2)	119 (16.3)	259 (35.5)	38.0
Male	3,251	1,382 (42.5)	658 (20.2)	1,211 (37.3)	41.0
Intellectual disability status					
IQ >70	2,038	787 (38.6)	398 (19.5)	853 (41.9)	43.0
IQ ≤70	1,057	617 (58.4)	188 (17.8)	252 (23.8)	34.0
IQ unknown	886	330 (37.2)	191 (21.6)	365 (41.2)	43.5
Race/Ethnicity					
Non-Hispanic white	2,063	935 (45.3)	399 (19.3)	729 (35.3)	39.0
Non-Hispanic black	859	342 (39.8)	196 (22.8)	321 (37.4)	42.0
Hispanic	730	313 (42.9)	129 (17.7)	288 (39.5)	40.0
**Total**	**3,981**	**1,734 (43.6)**	**777 (19.5)**	**1,470 (36.9)**	**40.0**

**Abbreviations:** ASD = autism spectrum disorder; IQ = intelligence quotient.

**FIGURE 2:**
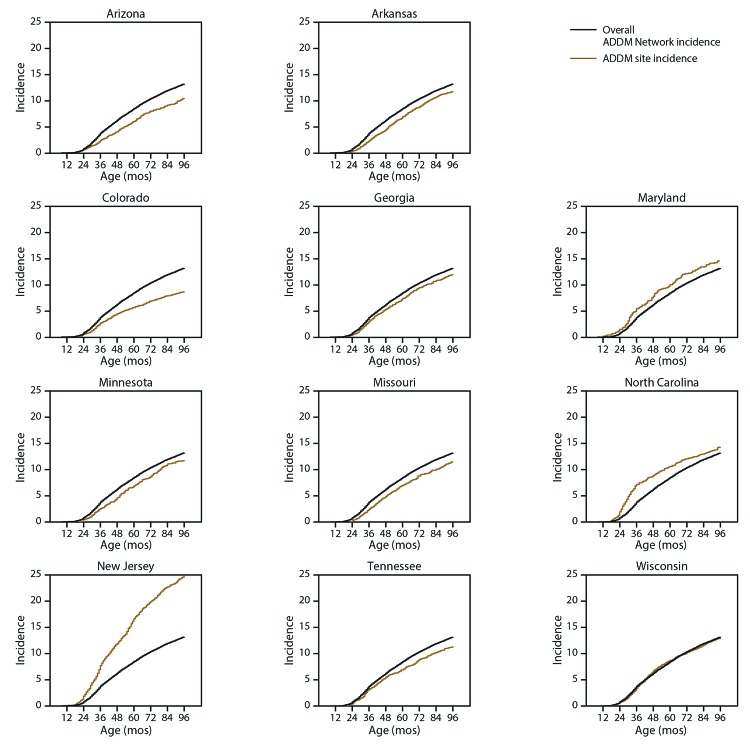
Cumulative incidence* of autism spectrum disorder diagnoses,^†^ by age and site — Autism and Developmental Disabilities Monitoring Network, 11 sites, United States, 2016 **Abbreviations:** ADDM = Autism and Developmental Disabilities Monitoring Network; ASD = autism spectrum disorder; ICD = International Classification of Diseases. * Per 1,000 children aged 8 years. ^†^ These data only include the portion of ADDM Network ASD cases with a documented diagnostic statement of ASD in the record. Children counted as ADDM Network cases without a documented ASD diagnosis (i.e., does not consider special education classifications or ICD codes) are not included.

Of the 5,108 children with ASD, 3,764 (74%) had an evaluation containing a statement of a clinical ASD diagnosis. Among those 3,764 children, the median age at ASD diagnosis was 51 months (range: 38 months [North Carolina] to 57 months [Arizona]) (Table 5). Children with ASD and IQ ≤70 had a median age at diagnosis of 44 months, whereas children with IQ >70 had a median age at diagnosis of 57 months. Among children with ASD and IQ ≤70, black children had an older median age at diagnosis than white children (48 versus 42 months). The cumulative incidence of ASD diagnoses indicates that community providers in New Jersey diagnosed more ASD cases by age 3 years than any other ADDM Network site, although the median age at diagnosis in New Jersey was the same as that of the overall ADDM Network (51 months) (Figure 2). The overall cumulative incidence of ASD diagnoses was 13.2 per 1,000 children by the time they turned age 8 years.

**TABLE 5:** Median age at earliest known autism spectrum disorder diagnosis, by intellectual disability status — Autism and Developmental Disabilities Monitoring Network, 11 sites, United States, 2016

Site/Characteristic	No. with ASD	All children with an ASD diagnosis*		Children with an ASD diagnosis and IQ ≤70		Children with an ASD diagnosis and IQ >70	
		No.	Median age (mos) at diagnosis	No.	Median age (mos) at diagnosis	No.	Median age (mos) at diagnosis
**Site**							
Arizona	282	193	57.0	45	51.0	127	59.0
Arkansas	606	489	56.0	204	49.0	267	63.0
Colorado	537	362	48.5	105	43.0	187	54.0
Georgia	456	306	55.0	116	50.5	138	60.0
Maryland	192	150	47.5	39	37.0	60	53.0
Minnesota	313	170	56.0	40	51.5	112	60.0
Missouri	213	194	56.0	13	68.0	37	71.0
New Jersey	1,036	844	51.0	162	44.0	431	53.0
North Carolina	489	280	38.0	95	30.0	156	49.0
Tennessee	405	307	51.0	112	41.5	142	59.0
Wisconsin	579	469	49.0	128	42.0	173	59.0
**Characteristic**							
Sex							
Female	938	649	51.0	211	43.0	281	56.0
Male	4,170	3,115	51.0	848	45.0	1,549	57.0
Race/Ethnicity							
Non-Hispanic white	2,613	1,950	51.0	468	42.0	1,064	58.0
Non-Hispanic black	1,070	781	53.0	295	48.0	283	57.0
Hispanic	909	674	51.0	196	44.0	310	55.0
**Total**	**5,108**	**3,764**	**51.0**	**1,059**	**44.0**	**1,830**	**57.0**

**Abbreviations:** ASD = autism spectrum disorder; IQ = intelligence quotient.

* Children with unknown IQ are included in the “all children with an ASD diagnosis” category but not the IQ-specific strata.

### Comparison with Prevalence of Community-Identified ASD

**FIGURE 3:**
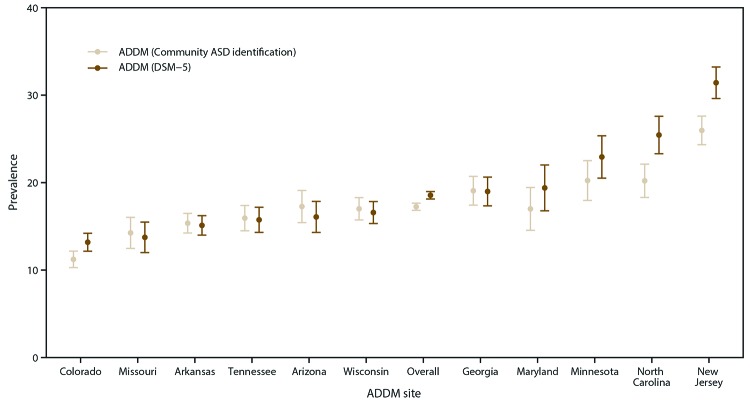
Comparison of autism spectrum disorder prevalence*^,†^ on the basis of *Diagnostic and Statistical Manual of Mental Disorders, Fifth Edition*, criteria and community-identified^§^ autism spectrum disorder prevalence, by site and overall — Autism and Developmental Disabilities Monitoring Network, 11 sites, United States, 2016 **Abbreviations:** ADDM = Autism and Developmental Disabilities Monitoring Network; ASD = autism spectrum disorder; DSM-5 = *Diagnostic and Statistical Manual of Mental Disorders, Fifth Edition*; ICD = International Classification of Diseases. * Per 1,000 children aged 8 years. ^†^ The dots represent prevalence estimates and the vertical lines bounded by bars represent the corresponding 95% confidence intervals. ^§^ Children are considered to be identified as having ASD by the community if they have an autism diagnosis stated in an evaluation, have been determined to meet autism eligibility in special education, or have been assigned an autism ICD code. Missouri, Colorado, and Wisconsin had limited access to information from educational records.

In addition to ASD diagnoses written in developmental evaluations, many children received an ASD classification in school (Supplementary Table 8, https://stacks.cdc.gov/view/cdc/85386), and certain children’s medical records contained ICD billing codes indicating ASD. The prevalence of all children with an ASD diagnosis, education classification, or ICD code was 17.2 per 1,000 children aged 8 years, approximately 7% lower than the ADDM Network estimate of 18.5 per 1,000 children aged 8 years (Figure 3). Although community-identified ASD prevalence was similar to ASD prevalence on the basis of the ADDM Network case definition, ADDM sites with higher prevalence also ascertained more children without an ASD diagnosis.

## Discussion

The latest ASD prevalence estimate, as measured by the ADDM Network, is 18.5 per 1,000 children aged 8 years in 2016. This is approximately 10% higher than the 16.8 prevalence estimate the ADDM Network reported in 2014 (*3*) and approximately 175% higher than (2.8 times) the first estimates reported by the ADDM Network in 2000 and 2002 (*4*,*5*). These changes could reflect differences in community practices for identifying ASD, changes in the data available to the surveillance system, or other unknown factors.

As with previous reports, observed ASD prevalence varies among ADDM Network sites. Community-level differences related to ASD diagnosis or classification for services correlate with ASD prevalence estimates; ASD prevalence and rank order of estimates across sites were similar to the prevalence of community-identified ASD. Previous analyses from the ADDM Network have shown a positive association between neighborhood socioeconomic status (SES) and ASD prevalence, which suggests ASD might be more readily identified in high-SES communities or among populations with good access to services (*16*). In this sense, the ADDM Network prevalence estimates could be used to support efforts to improve ASD diagnosis for lower-SES groups in the community.

### Timely Evaluations and Age at First ASD Diagnosis

For the first time since ADDM began, no statistically significant difference was found in the overall ASD prevalence among black and white children. This diminishing disparity in ASD prevalence might signify progress toward earlier and more equitable identification of ASD. Although black children with ASD were more likely than white children to have an intellectual disability and children with intellectual disability were more likely to be evaluated early, black children were still less likely than white children to be evaluated by age 36 months. In addition, among children with intellectual disability, the median age at ASD diagnosis was 6 months later for black than for white children. Further study is needed to identify community-level barriers to timely evaluation and diagnosis of ASD so that treatments can be delivered as early as possible. Examining differences between communities with earlier and later ASD identification might reveal successful practices or policies that could be implemented in other communities. CDC’s “Learn the Signs. Act Early.” initiative works with Act Early Ambassadors who support state and territorial or national efforts to improve early identification of developmental disabilities and promote the integration of developmental monitoring in systems that serve children and their families (https://www.cdc.gov/ncbddd/actearly).

Although early diagnosis of ASD is a major public health goal and one of the *Healthy People 2020* objectives (https://www.healthypeople.gov/2020/default), the median age at first ASD diagnosis has changed little over the course of ADDM Network reporting. However, this metric might not fully capture community progress toward early identification and could mask improvement. For instance, the median age at ASD diagnosis will increase if the community begins diagnosing more ASD among children at older ages who in previous years would not have received an ASD diagnosis by age 8 years. An absolute metric, such as cumulative incidence, might reveal advances in early identification over previous cohorts, as shown in the Early ADDM Network report (*28*).

### Comparison with Other Autism Data Systems

The National Survey of Children’s Health (NSCH) and the National Health Interview Survey (NHIS) are two U.S. nationally representative surveys that measure ASD prevalence by asking parents and caregivers if a doctor or health professional told them that their child has ASD. The 2016 NSCH and the 2015–2017 NHIS both estimated ASD prevalence at 25 per 1,000 children aged 3–17 years (*13*,*29*). Important differences exist between the national surveys and the ADDM Network that warrant consideration when comparing prevalence estimates. The surveys rely on parent-reported ASD diagnoses among children aged 3–17 years in a nationally representative sample, whereas the ADDM Network uses documented information from qualified professionals and monitors ASD among children aged 8 years in participating communities. These data sources might be used in complementary ways; for instance, most ADDM Network sites indicate lower ASD prevalence than the survey estimates, which might indicate a need for improved ASD identification in those communities. To facilitate comparisons between different data systems, CDC developed an interactive website that presents U.S. state-based ASD prevalence data from four data systems (ADDM Network, NSCH, Medicaid, and special education) (https://www.cdc.gov/ncbddd/autism/data/index.html).

ASD prevalence reports from other countries provide information about aspects of ASD that are not measured in the United States and allow for comparisons with the ADDM Network. In 2018, Canada released the first National Autism Surveillance System (NASS) report (*30*). NASS monitored ASD among 1.9 million children aged 5–17 years across all Canadian provinces in 2015 and relied on existing ASD diagnoses to ascertain cases. The overall ASD prevalence was 15.2 per 1,000 children with province estimates ranging from 8.0 to 17.5 per 1,000, which was slightly lower than estimates from the ADDM Network. NASS data indicated that 28% of children with ASD received the diagnosis after age 8 years. A study using linked registry data from Denmark revealed an even greater proportion of children with ASD diagnosed after age 8 years (*31*). Danish children born in 2008 had a cumulative incidence of diagnosed ASD of approximately 12 per 1,000 children by age 8 years (comparable to the cumulative incidence of ASD diagnoses among children aged 8 years in the ADDM Network) (Figure 2); among older Danish cohorts, cumulative incidence was as high as 28 per 1,000 by age 15–16 years.

## Limitations

The findings in this report are subject to at least four limitations. First, the ADDM Network methods rely on the quality and completeness of existing documents to ascertain cases. Sites without access to education records for large portions of their population might not be ascertaining children (particularly black or Hispanic children) (*32*) who only receive services for ASD at school. Second, record completeness is also important for documenting when ASD was first diagnosed in a child, whether the child had IQ testing, and when a child was first evaluated. Reduced access to records, incomplete records, or both, could lead to an underestimate of the number of children identified as having ASD. Third, sites participating in the ADDM Network are funded through a competitive process and are encouraged to include diverse communities; however, the resulting sites are not nationally representative and do not generate nationally representative ASD prevalence estimates. Finally, geographic coverage of the ADDM Network has changed over time, complicating interpretation of temporal trends.

## Future Directions

The ADDM Network will continue data collection for the 2018 and 2020 surveillance years. Sites in 11 states will monitor ASD prevalence among children aged 4 and 8 years. Five sites have initiated a new activity to describe outcomes for children aged 16 years who were initially ascertained by the ADDM Network at age 8 years. To provide the most comprehensive information on how communities identify, serve, and support persons with ASD, the ADDM Network increasingly will focus on community indicators of ASD identification, disparities in service use, and co-occurring conditions among persons with ASD.

## Conclusion

These findings from the 2016 ADDM Network indicate considerable variability in ASD prevalence across communities and higher ASD prevalence than previous estimates from the ADDM Network. For the first time, no overall difference in ASD prevalence between black and white children was reported, although disparities in early intervention and identification persist for black children. ASD prevalence among Hispanic children continues to be lower than among white or black children. Black and Hispanic children with ASD were evaluated at older ages than white children and were more likely to have intellectual disability. Black children with intellectual disability and ASD also received diagnoses at older ages than did white children with intellectual disability and ASD, which might limit opportunities to receive services that could improve their outcomes and quality of life. ASD continues to be a public health concern; the latest data from the ADDM Network underscore the ongoing need for timely and accessible developmental assessments, educational supports, and services for persons with ASD and their families.
