# *Erratum:* Vol. 69, No. SS-4

**DOI:** 10.15585/mmwr.mm6916a4

**Published:** 2020-04-24

 In the Surveillance Summary “Prevalence of Autism Spectrum Disorder Among Children Aged 8 Years—Autism and Developmental Disabilities Monitoring Network, 11 Sites, United States, 2016,” errors occurred in the Abstract and Table 2 and Table 3.

 On page 1 in Results, the percentage of girls with intellectual disability should have been **39%.** On page 11, the second footnote of Table 2 should have referenced **Supplementary Table 7**. On page 11, the fourth column header in Table 3 should have been IQ **≤70**.

